# Exploring the link between empathy, stress, altruism, and loneliness in university students during the COVID‐19 pandemic: A cross‐sectional study

**DOI:** 10.1002/brb3.70049

**Published:** 2024-09-25

**Authors:** Anders Larrabee Sonderlund, Sonja Wehberg, Elisabeth Assing Hvidt

**Affiliations:** ^1^ Department of Public Health University of Southern Denmark Odense Denmark

**Keywords:** Empathy, stress, loneliness, mental health, vicarious stress

## Abstract

**Background:**

Empathy has been associated with a range of positive outcomes, including social connection, pro‐social behavior, and mental health. Nonetheless, acknowledging the multidimensional aspects of empathy, budding research indicates that sometimes empathy may precipitate negative health outcomes.

**Aim:**

In the present paper, we explore the extent to which the multidimensional aspects of empathy—as measured by the Interpersonal Reactivity Index—may relate to the experience of increased stress during the COVID‐19 pandemic. We also examine potential behavioral moderators (altruism) and social mediators (loneliness) of any such effect.

**Method:**

We conducted a cross‐sectional survey study of 2595 Danish university students and implemented linear regression analyses to determine the relationships between our key variables.

**Results:**

In both crude and adjusted regression models, our findings indicate positive associations between the IRI subscales Fantasy, Empathic Concern, and Personal Distress on self‐reported stress and loneliness. Perspective Taking was marginally and inversely associated with stress but not loneliness. Altruism did not moderate these associations, but loneliness did mediate the effects of Fantasy, Empathic Concern, and Personal Distress on stress.

**Discussion:**

Our results emphasize the importance of conceptualizing empathy in multi‐dimensional terms. Further, our study highlights the potential negative health consequences of empathy in certain settings. These results may help focus future research in this area and feed into mental health interventions.

## Graphical abstract

Our results support the idea that empathy is a multidimensional construct that may support and undermine individual mental health. Operationalizing empathy in terms of the Interpersonal Reactivity Index, we find that in times of social isolation different types of empathy increase the experience of individual loneliness and, by extension, stress.

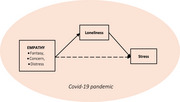

## 1 INTRODUCTION

Empathy is generally considered a positive characteristic and a marker of social and emotional intelligence (Hojat, 2016). The ability to truly intuit and appreciate someone else's thoughts and feelings, motives and context has been linked to increased compassion and altruistic behaviors in a variety of settings (Batson et al., 2004; Petrocchi et al., 2021; Rose et al., 2022; Vardy & Atkinson, 2019; Vollhardt, 2009). There is also evidence that empathy may confer individual health benefits—specifically by protecting the individual from a range of mental illnesses ‐ including anxiety and depression ‐ by facilitating emotion regulation, resilience, and social connection (Haslam et al., 2018; Schipper & Petermann, 2013; Thompson et al., 2019; Zaki, 2020). These findings, however, are somewhat complicated by budding research that suggests that while empathy is often adaptive, it is also a complex characteristic to which there may be several health‐related negative consequences (Harvey, 2003; Stosic et al., 2022). Specifically, in certain situations empathy might become a health risk by causing different types of stress, including *vicarious stress* and loneliness (a known stressor) (Beadle et al., 2012; Holaday et al., 2022; Larrabee Sonderlund et al., 2019; Shu et al., 2017; Wagaman et al., 2015; Weber et al., 2022).

Vicarious stress occurs when the individual experiences secondhand stress through empathic engagement with someone else's trauma or adversity (Huggard & Unit, 2013; Jennings & Min, 2023). Research suggests that vicarious stress affects the individual's psychology and physiology in much the same way as firsthand stress, and thus represents a serious psychophysiological health risk if experienced regularly or chronically (Larrabee Sonderlund et al., 2019). Common symptoms include mental fatigue, anxiety, and depression (Regehr et al., 2002; Van Mol et al., 2015). In turn, these psychological outcomes may contribute to increased risk of noncommunicable diseases (including cardiometabolic disease and cancer) through health‐risk behavior and allostatic load (i.e., the chronic activation of the hypothalamic‐pituitary‐adrenal axis stress response) (Juster et al., 2010). While people in caregiver‐ or support‐based occupations (e.g., health professionals (Austin et al., 2009; Huggard & Unit, 2013; Kerman et al., 2022; Lewis et al., 2022), psychologists (Aafjes‐van Doorn et al., 2020; Stebnicki, 2007), social workers (Newell & MacNeil, 2010)) might be particularly exposed to this type of stress by virtue of their profession, it can also manifest in everyday supportive relations among friends and family, colleagues and acquaintances (Engert et al., 2014; Russell & Brickell, 2015). To this point, the general public in many countries experienced vicarious stress at the height of the COVID‐19 pandemic as people empathized with their families, friends, and fellow citizens through social isolation, job‐loss, illness, and death (Li et al., 2020; Liu & Liu, 2020; Zhao et al., 2021).

Empathy might also lead to stress by making the individual more vulnerable to the effects of loneliness. This is more explicitly relevant in times of social isolation—for example, during the COVID‐19 pandemic when lockdowns and social distancing measures were implemented for extended periods of time (Kalinowski et al., 2022). Past studies indicate that because empathy is an inherently social emotion that encourages and facilitates social connection (an important buffer against stress and related health outcomes) (Alam et al., 2016; Huo et al., 2020; Larrabee Sonderlund et al., 2019), people high in empathy may rely on social interaction and support for general well‐being more than people low in empathy. Given this, when social interaction is restricted for a period of time (as it was in many countries during the COVID‐19 pandemic), empathy may actually increase the experience of loneliness—a known source of stress (Beadle et al., 2012; Holaday et al., 2022; Larrabee Sonderlund et al., 2019; Weber et al., 2022).

The research on the implications of empathy for individual health and well‐being is thus somewhat inconsistent, with some studies reporting benefits for mental health and resilience and others showing the opposite. These diverging results probably reflect the multifaceted and mercurial nature of empathy as a psychological phenomenon that may be expressed and internalized in myriad ways depending on multiple factors (Batchelder et al., 2017; Stosic et al., 2022). For instance, research that differentiates between emotional and cognitive empathy suggests that vicarious stress might be triggered by the former empathic style to a greater degree than the latter (Caringi & Pearlman, 2009; Harvey, 2003; Nummenmaa et al., 2008). That is, engaging with other people's adversity instinctually and emotionally might expose the empathizer to a more visceral and personal experience of secondhand stress (Harvey, 2003; Nummenmaa et al., 2008). By contrast, a more reasoned and cognitive understanding of someone else's hardship (as distinctive from one's own) might afford the perceiver some protection from the experience of associated stress (Nummenmaa et al., 2008; Park et al., 2021). For this reason, a more cognitive style of empathy is sometimes taught and cultivated among health professionals and social workers to mitigate stress and burnout and so they can remain composed and effectual in stressful situations (Hunt et al., 2019; Pérez‐Fuentes et al., 2020; Teding van Berkhout & Malouff, 2016).

Adding further complexity, there is also evidence that certain types of empathy may galvanize altruistic behavior and thus buffer the experience of stress (Batson, 2017; DeSteno, 2015; Enelamah & Tran, 2020; Farrelly & Bennett, 2018; Persson & Kajonius, 2016). Past research indicates that if the individual can act on their feelings of (emotional) empathy by engaging in compassionate and altruistic behavior to alleviate another's suffering, then this may mitigate the ill effects of any empathy‐related stress (Cosley et al., 2010; de Lima Junior et al., 2022; Dulin & Hill, 2003; Kornilaki, 2022; Miller et al., 2015; Schwartz et al., 2003; Seppala et al., 2013). In other words, this evidence suggests that the stress of empathizing with someone's plight  may be assuaged by actively trying to improve that person's situation—for example by offering emotional support or doing relevant charity work.

Acknowledging the inherent complexity of empathy and the ambiguity of the existing evidence base, more knowledge is needed about when and how the multidimensional aspects of empathy might confer explicitly negative health implications. In this study, we focus specifically on stress.

### 1.1 Rationale and aims

Operationalizing and measuring empathy scientifically require an inclusive conceptualization that accounts for the multifaceted nature of this construct. To this end, the Interpersonal Reactivity Index (IRI) represents one of the more comprehensive empirical measurement tools. The IRI was developed by Davis (1980a) and comprises a reliable and validated index of four basic social, emotional, and cognitive aspects of empathy. *Fantasy* (FS) is the tendency to transpose oneself into a given fictional situation and experience feelings and thoughts that are consistent with that setting—for example while engrossed in a novel or movie. *Perspective taking* (PT) concerns the ability to understand a situation from someone else's point of view. *Empathic concern* (EC) describes sympathy and worry for others’ adversity or misfortune as well as feelings of warmth and compassion toward others. Finally, *Personal distress* (PD) refers to intense feelings of stress and anxiety that are triggered by seeing or learning of others’ calamity and hardship. The IRI thus represents a rather fluid cognitive‐emotional spectrum of empathy. At one end, PD may be considered a measure of instinctive and felt emotional empathy, whereas PT at the other end denotes a more rational, distanced, and analytical appreciation of someone else's experience. EC and FS fall in between these two extremes as aspects of empathy that span both emotional and cognitive dimensions (Hojat, 2016; Assing Hvidt et al., 2023).

In the following sections, we report the results of a survey study into the association between empathy (as measured by the IRI) and psychological stress (as measured by PSS‐10) during the COVID‐19 pandemic among students at four Danish universities. In line with the literature discussed above, we explore the extent to which different facets of empathy may expose the individual to, or protect them from, loneliness and different types of stress. We also delve into the potential mechanisms of these associations, specifically examining the aforementioned indication that empathy motivates altruism (Batson, 2017; DeSteno, 2015; Enelamah & Tran, 2020; Farrelly & Bennett, 2018; Persson & Kajonius, 2016) and that engaging in altruistic behavior may help the individual cope with empathy‐related vicarious stress (Cosley et al., 2010; de Lima Junior et al., 2022; Dulin & Hill, 2003; Kornilaki, 2022; Miller et al., 2015; Schwartz et al., 2003; Seppala et al., 2013). We argue that explicating the process of how empathy benefits versus compromises individual mental health (i.e., stress) might inform resilience‐building initiatives and therapeutic interventions that target mental health in a variety of settings.

## 2 METHOD

### 2.1 Participants and procedure

The study was conducted with a cross‐sectional survey design and comprised 1st‐, 3rd‐, and 5/6th‐year students from four universities in Denmark. The survey was programmed and made accessible on the SurveyXact survey system. Data collection was completed in December 2020. As such, data collection took place during the height of social distancing measures in Denmark. All university classes were moved online, and campuses were closed. Bars, restaurants, cinemas, sports clubs, gyms, and other similar amenities were also closed, and the public was advised to not engage in face‐to‐face contact with people outside of their household. A total of 14,702 eligible students was invited to participate in the study via an information letter sent to students’ university email addresses and/or their personal “e‐boks” (e‐box—a secure digital mail system that is mandatory for Danish citizens and permanent residents and used exclusively for communication between individuals and government, healthcare, banks, etc.). The letter contained a plain language statement about the voluntary nature of the study, what participation would involve, GDPR compliance information, contact details of the principal investigators, and confirmation that the study was approved by the university institutional review board (journal #10.181). Students consented to participate by checking a box confirming their informed consent. They were then redirected to the online survey. Background data (including date of enrolment, current year of study, and major) for all participants was accessed through university enrolment lists and linked with participant responses at the individual level using participants’ social security number.

### 2.2 Survey measures

In addition to an initial set of four demographic questions about participant sex, age, marital/partner status, and cohabitation, the survey comprised validated items and scales that tapped respondents’ self‐reported empathy, compassion toward others and self, altruism, attachment style, coping style, life values, as well as perceived stress, quality of life, mental burden associated with the COVID‐19 pandemic, including perceived loneliness, and self‐reported religiosity. The survey comprised 122 items and took approximately 20 min to answer. For the purposes of the present study, we focused on the variables described below. To assess whether common methods bias was present in our data, we conducted a Harmon's one‐factor test for common methods bias. The total variance extracted by one factor was 15.38% and thus did not breach the 50% threshold for common methods bias.

#### 2.2.1 Primary outcome

*The Perceived Stress Scale (PSS‐10)*. The PSS‐10 was used to measure participant stress levels and represents our primary outcome. The PSS‐10 was originally developed by Cohen et al. (1983) and has been translated and validated in multiple countries, including Denmark (Eskildsen et al., 2015). The 10‐item scale assesses the extent to which individuals have experienced life as unpredictable, uncontrollable, and burdensome in the previous month. Answers were scored on five‐point Likert scales ranging from 0 = Never to 4 = Very often. The total PSS‐10 score was calculated by reverse scoring items 4, 5, 7, and 8, and then averaging across all items. Higher scores indicated higher levels of experienced stress. The PSS‐10 exhibited high internal reliability (Cronbach's *α* = .890).

#### 2.2.2 Secondary outcome

*Loneliness during the COVID‐19 pandemic*. In addition to the PSS‐10, we also created a single‐item measure of the degree to which participants had felt lonely as a result of the pandemic (“*To what extent has the COVID‐19 pandemic made you feel lonely?*”). Answers were scored on five‐point Likert scales ranging from 0 = Not at all, to 4 = Very much so.

#### 2.2.3 Independent variables

*Interpersonal Reactivity Index (IRI)*. The IRI comprises 28 items that measure the four previously mentioned dimensions of empathy (Davis, 1980b): (1) *Fantasy*, (2) *Perspective taking*, (3) *Empathic concern*, and (4) *Personal distress*. Seven items tap each dimension and are phrased as descriptive statements (e.g., *“I sometimes try to understand my friends better by imagining how things look from their perspective”*) and respondents indicate on five‐point Likert scales the degree to which each statement describes them well or not (0 = Does not describe me well, 4 = Describes me very well). Participants receive an average score on each of the four empathy domains. The IRI subscales had good to high internal reliability (Cronbach's *α* Fantasy = .808; Empathic Concern = .803; Perspective Taking = .682; Personal Distress = .822). As the distinct IRI components have been validated and used in multiple previous studies, additional validation of these components was not necessary (Chrysikou & Thompson, 2016; De Corte et al., 2007; Gupta et al., 2022).

*The Generative Altruism Scale (GAIS)*. The GAIS is a four‐item scale that assesses attitudinal altruism. Here, altruism is operationalized as the individual's commitment to helping others in need and doing so without expecting any reward or other benefit (e.g., *“When I see someone suffering, I try to find ways to help him/her”*). Thus, the GAIS items focus exclusively on the individual's self‐perceived likelihood that they would engage in altruistic actions rather than tapping any beliefs, attitudes, or ideology that may motivate such behavior. The GAIS is scored on four‐point Likert scales ranging from 1 = Strongly disagree to 4 = Strongly agree, which are then averaged to create an overall score. The GAIS exhibited high internal reliability (Cronbach's *α* = .820).

#### 2.2.4 Covariates

We also included the covariates of participant age, sex, marital/partnership status (single, partner, married), and household (single, with spouse/partner, with others).

### 2.3 Analytic strategy

We implemented a four‐step analytic strategy. First, we generated descriptive statistics for each of our key variables and covariates, including frequencies, means, and bivariate Pearson's correlations. Second, exploring the association between empathy and stress, we regressed our primary outcome variable (stress—PSS‐10) onto the individual components of the IRI in both crude and adjusted (for covariates) regression models. Third, we assessed whether any or all of these associations were moderated by altruism (the GAIS). Fourth, in line with existing research (Alam et al., 2016; Beadle et al., 2012; Kalinowski et al., 2022) that empathy may be associated with increased loneliness (a stressor) in times of social isolation (i.e., the COVID‐19 pandemic), we explored the extent to which loneliness mediated an effect of empathy on stress. The mediation analysis was conducted using the PROCESS macro (model 4) with bootstraps set at 5000 samples, generating 95% confidence intervals by sorting the lowest to the highest of bootstrap samples. The bootstrap method is based on regression analysis with direct and indirect effects derived from two linear models. One estimates the mediator *M* from the exposure *X*: *M* = *i_M_* + *a*_1_ *X* + *e_M_*. The other estimates the outcome *Y* from both *X* and *M*: *Y* = *i_Y_* + *c'X* + *b_1_M* + *e_Y_*. Here, *i_M_* and *i_Y_* are regression constants, *e_M_* and *e_Y_* are errors in the estimations of *M* and *Y*, and *a*, *b*, and *c’* are regression coefficients for *X* predicting *M*, *M* predicting *Y*, and *X* predicting *Y*, respectively. The indirect effect of *X* on *Y* through mediator *M* is estimated as the product of the effect of *X* on *M* and the effect of *M* on *Y* (*a*_1_ *b*_1_) (Hayes, 2012, 2022). All analyses were conducted using IBM SPSS Statistics v28.0.1.0 (142) and mediation results were reported according to the AGReMA statement (Lee et al., 2021).

## 3 RESULTS

### 3.1 Descriptive statistics

#### 3.1.1 Demographics

A total of 2595 people participated in the study (a response rate of 18%). The sample comprised 65.1% women and 34.9% men. Participant age ranged from 18 to 64 years, with a mean of 24 years (*SD* = 2.63). In terms of relationship status, 46.4% had a partner, 6.1% were married, and 47.4% were single. Additionally, 31.7% lived alone, 36.3% lived with a partner, and 32.0% lived with others.

##### Frequencies, means, and correlations

Table 1 presents frequency and mean statistics for each of our key variables. Participants scored significantly higher than the scale midpoint on the FS, EC, and PT subscales (Table 1). On PD, however, they scored significantly lower. Similarly, on each of the other variables, participants scored lower than the scale midpoint, indicating relatively low levels of stress, altruism, and loneliness.

**TABLE 1:** Means, SDs, and scale midpoints for each key variable.

Variable	Mean (SD)	Scale mid‐pt	Minimum	Maximum
IRI Fantasy	2.56 (0.79)*	2.00	0.00	4.00
IRI Empathic Concern	2.84 (0.68)*	2.00	0.00	4.00
IRI Perspective Taking	2.70 (0.61)*	2.00	0.00	4.00
IRI Personal Distress	1.47 (0.74)*	2.00	0.00	4.00
GAIS Altruism	1.18 (0.45)*	2.50	0.00	3.00
Pandemic loneliness	2.69 (1.24)*	3.00	1.00	5.00
PSS stress	18.28 (0.71)*	20.00	0.00	40.00

*Different from scale midpoint at *p* <.05.

The interrelationships between our predictor variables (the IRI subscales) and our outcomes are displayed in Table 2. FS, EC, and PD were associated with increased stress. PT, however, was not significantly related to stress. Further, FS, EC, and PT correlated positively with altruism, while PD was inversely associated with this outcome. Finally, loneliness correlated positively with stress as well as with FS, EC, and PD, but did not correlate with PT at a statistically significant level.

**TABLE 2:** Bivariate correlations between each of our key variables.

Variable	1	2	3	4	5	6
IRI Fantasy	–					
IRI Perspective Taking	.194**	–				
IRI Empathic Concern	.445**	.386**	–			
IRI Personal Distress	.179**	–.147**	.143**	–		
Altruism	.211**	.390**	.439**	–.161**		–
Pandemic loneliness	.155**	.005	.115**	.175**	.068**	
Stress	.190**	–.067**	.205**	.425**	.052**	.399**

**p <.01.

##### Regression analyses

Results from our regression analyses showed that FS (*β* = .19, 95% CI 0.15, 0.23), EC (*β* = .21, 95% CI 0.17, 0.24), and PD (*β* = .43, 95% CI 0.39, 0.46) were associated with increased stress. These associations held up when controlling for covariates (Table 3). PT correlated negatively but marginally with stress in crude (*β* = –.07, 95% CI 0.11, 0.03) and adjusted models.

**TABLE 3:** Crude (Model 1) and adjusted (Model 2) linear regression models.

Model 1					Model 2			
	*β*	*t*	95% LCI	95% UCI	*β*	*t*	95% LCI	95% UCI
IRI Fantasy	.19*	9.85	0.15	0.23	.14**	7.16	0.10	0.18
Sex					.20**	10.23	0.16	0.24
Age					–.01	–0.50	–0.05	0.03
Partner					.09**	4.26	0.05	0.12
Living situation					–.04*	−2.06	–0.08	–0.00
IRI Perspective Taking	–.07*	−3.44	–0.11	–0.03	–.08*	−4.21	–0.12	–0.04
Sex					.24	12.63	0.20	0.28
Age					–.01	–0.65	–0.05	0.03
Partner					.08	4.14	0.04	0.12
Living situation					–.04	−1.87	–0.07	0.00
IRI Empathic Concern	.21*	10.67	0.17	0.24	.15**	7.24	0.11	0.19
Sex					.18	8.85	0.14	0.22
Age					–.02	−1.17	–0.06	0.02
Partner					.09	4.53	0.05	0.13
Living situation					–.04	−2.29	–0.08	–0.01
IRI Personal Distress	.43*	23.93	0.39	0.46	.39**	21.80	0.36	0.43
Sex					.16	8.94	0.13	0.20
Age					.02	1.20	–0.01	0.06
Partner					.06	3.22	0.02	0.10
Living situation					–.03	−1.40	–0.06	0.01

*p ≤.05.

**p <.01.

To assess whether altruism moderated the observed associations between empathy and stress, we added altruism to our regressions for FS, EC, and PD. Altruism added little to the models for FS (*β* = .01, 95% CI –0.53, 0.76) and EC (*β* = –.03, 95% CI −1.19, 0.20), but contributed significantly to the models for PD (*β* = .11, 95% CI 1.18, 2.36) and PT (*β* = .08, 95% CI 0.60, 1.97). Probing the moderation effects further, we added PDxAltruism and PTxAltruism interaction terms to our fully adjusted regression models for PD and PT (Model 2). This produced nonsignificant results for both PT (PtxAltruism *β* = –.00, 95% CI −1.07, 0.84) and PD (PDxAltruism *β* = –.03, 95% CI −1.28, 0.19) indicating no moderation.

##### Mediation analyses

**FIGURE 1:**
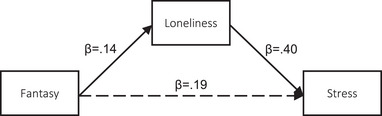
Pathway diagram for the association between IRI Fantasy and stress via loneliness.

**FIGURE 2:**
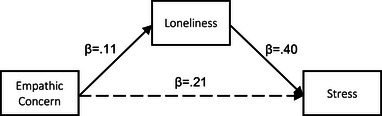
Pathway diagram for the association between IRI Empathic Concern and stress via loneliness.

**FIGURE 3:**
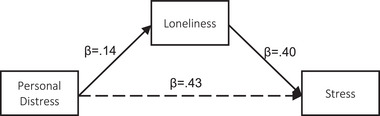
Pathway diagram for the association between IRI Personal Distress and stress via loneliness.

We next assessed the extent to which loneliness mediated the associations between the IRI dimensions and stress. Statistics for the individual pathways leading from each IRI component to the mediator (loneliness), and the mediator to the outcome (stress) are provided in Table 4. All pathways were statistically significant except for PT‐loneliness. Indirect effects (IE—i.e., mediation) for FS, EC, and PD are presented in Table 5 and depicted in Figures 1, 2, 3. Results from crude mediation models indicated that the positive associations between FS, EC, and PD and stress were mediated by loneliness (FS: IE = 0.56, SE = 0.07, 95% CI 0.42, 0.71) (EC: IE = 0.48, SE = 0.09, 95% CI 0.32, 0.66) (PD: IE = 0.60, SE = 0.08, 95% CI 0.46, 0.75). These results persisted in fully adjusted models (Table 5).

**TABLE 4:** Fully adjusted statistics for IV to mediator, and mediator to DV pathways.

Path *a*_1_: IV to mediators						
IV	Mediator	*β*	*t*	95% LCI	95% UCI	
IRI Fantasy	Loneliness	.137*	7.009	0.154	0.273	
IRI Perspective Taking	Loneliness	–.003	–0.165	–0.083	0.070	
IRI Empathic Concern	Loneliness	.107*	5.185	0.121	0.268	
IRI Personal Distress	Loneliness	.140*	7.233	0.171	0.298	
**Path *b*_1_: Mediator to outcome**						
Mediator	Outcome	*β*	*t*	95% LCI		95% UCI
Loneliness	Stress	.399*	22.180	2.233		2.666

*p <.05.

**TABLE 5:** Fully adjusted indirect effects of IRI components on stress via loneliness.

IV	DV	Mediator	IE	Boot SE	95% LCI	95% UCI
IRI Fantasy	Stress	Loneliness	0.471	0.073	0.331	0.618
IRI Empathic Concern	Stress	Loneliness	0.488	0.089	0.317	0.663
IRI Personal Distress	Stress	Loneliness	0.478	0.074	0.335	0.623

## 4 DISCUSSION

This study suggests associations between different dimensions of empathy and the experience of stress. Specifically, our results show that FS, PD, and EC were associated with increased stress during the COVID‐19 pandemic. This is consistent with other research linking the capacity for empathy with vicarious stress (Huggard & Unit, 2013; Jennings & Min, 2023). As one might expect, this association was strongest for PD, which may be construed as the epitome of vicarious stress (Konrath et al., 2011). Similarly, FS has previously been associated with emotional vulnerability, so the positive correlation with stress aligns with existing evidence as well (Davis, 1983b). In terms of EC, however, these findings are somewhat more notable. EC has previously been conceptualized as a particularly adaptive dimension of empathy that drives compassion and galvanizes altruistic intent, thus increasing feelings of self‐efficacy and self‐esteem, and buffering stress (Davis, 1980b; Haslam et al., 2018; Konrath et al., 2011). Our results diverge somewhat from this literature, though, by showing a positive association between EC and stress. We theorize that this may be due to the unique setting in which our study was conducted which was characterized by various and wide‐reaching pandemic social distancing interventions. These measures likely inhibited hands‐on altruistic behavior and thus may have neutralized an important stress‐buffering aspect of empathic concern, replacing it instead with feelings of helplessness and distress. The fact that our results showed no moderation of altruism in the association between any aspect of empathy and stress may reflect participants’ restricted ability to bear their altruistic intent to fruition due to social distancing measures.

Our results also contribute the novel finding that loneliness may mediate the effect of FS, PD, and EC on stress. This aligns with past research that conceptualizes empathy as a beneficial aspect of social cognition that arises from (and helps satisfy) the basic human need for connection, community, and belonging (Decety et al., 2012). In fact, there is evidence of a strong positive correlation between empathy and social connectedness (Jetten et al., 2012; Riley et al., 2022; Wölfer et al., 2012). This association is often attributed to the idea that empathy facilitates positive social connections by enabling mutual understanding, compassion, and respect between individuals—key ingredients for the development of any meaningful relationship (Jetten et al., 2012). Adjacent research shows how supportive social connections represent a central source of individual resilience to stress and overall mental well‐being (Haslam et al., 2018; Larrabee Sonderlund et al., 2017). Taken together, this research may indicate that people high in empathy rely heavily on their social world and connections for psychological resilience. However, if this social connectedness and interaction is disrupted—for instance by prolonged social distancing measures—this may result in feelings of social disconnection, isolation, and loneliness, and in turn undermine the individual's psychological defenses against the ill effects of stress (Beadle et al., 2012; Davis, 1980b; Haslam et al., 2018; Holaday et al., 2022; Huo et al., 2020).

Finally, PT correlated inversely (and marginally) with stress and nonsignificantly with loneliness, indicating a clear divergence of this dimension of empathy from the other three (FS, PD, EC). This is consistent with other studies, noted in the introduction, that have found that more cognitive and reasoned empathic reactivity may provide some level of emotional separation between the empathizer and the object of their empathy., Presumably this offers some protection from any vicarious stress. PT has also been associated with high self‐esteem and self‐reliance, as well as with low social dysfunction (including loneliness). This may explain the lack of correlation with loneliness‐related stress (Davis, 1983a, 1983b).

### 4.1 Implications

Our findings have several theoretical and practical implications. Firstly, our results support the idea that empathy is a complex multidimensional construct rather than a unidimensional one. This is important for future research in this area in terms of the specificity of research questions (i.e., which aspects of empathy are in focus), the empirical operationalization of empathy (multidimensional measures over global ones), as well as the underlying mechanisms by which empathy may impact on a given outcome of interest. As a case in point, the four IRI subscales included in our study were related to stress to varying extents and by two different pathways (vicarious engagement and loneliness). This finding attests to the notion that the propensity for empathy is not merely a general capacity, but rather manifests in qualitatively distinctive multitudinal psychological terms (i.e., FS, EC, PT, PD).

In more applied terms, a key contribution of our study centers on the finding that while there are many benefits to empathy, there are sometimes also negative repercussions. These downsides appear to be context‐dependent, relating primarily to vulnerabilities (vicarious stress, loneliness) that arise in times of pervasive suffering and/or social isolation. While the context for our findings comprises a rather unique setting (i.e., a global pandemic), the mechanisms by which empathy may have harmful individual consequences may also be relevant in other, more common contexts. These may include war zones, natural disasters, widespread poverty and famine. Other everyday settings may include individual life situations such as those defined by certain occupations (e.g., healthcare workers, mental health professionals, etc.) or other circumstances (e.g., incarceration, hospitalization) that expose the individual to extensive and persistent human adversity and/or social disruption. As such, our findings might help specify preventive interventions targeting stress and stress‐related outcomes (e.g., burnout, anxiety, depression) in people and populations exposed to these types of situations. Indeed, emerging research focuses on the extent to which PT can be taught to people in stressful healthcare professions as a way to protect their mental health and avoid burnout (Hunt et al., 2019; Pérez‐Fuentes et al., 2020; Teding van Berkhout & Malouff, 2016).

### 4.2 Strengths and limitations

The present study has several strengths. These include not only the large sample size, but also the fact that our sample comprised Danish university students. As a disproportionate number of studies in this area are conducted in North American and British populations, our results provide a much‐needed international perspective. Further, and as noted above, another strength relates to the multidimensional operationalization of empathy and the use of the IRI, which adds important nuance and qualifying context to previous studies on the nature and consequences of empathy. Finally, our mediation results also provide novel insight into the underlying mechanisms by which empathy may be associated with mental health outcomes. Additionally, these analyses were conducted using a statistically robust bootstrapping methodology that has gained traction in recent years as a rigorous alternative to more traditional methods (e.g., the Sobel test) (Hayes, 2012).

In addition to these strengths, there are also several limitations to our study. As our results are based on a cross‐sectional design, we cannot ascertain cause and effect. Even though we formulate the relations between our key variables within a mediation set‐up, which implies a certain order of effects and strongly resembles a causal model, without controls and temporal separation between our variables, we can only ever estimate associations. Given the nature of empathy as a characteristic that gradually develops over the life course (Uzefovsky & Knafo‐Noam, 2016), it is a tricky thing to manipulate in an experimental design. Future research, however, may implement longitudinal assessments of people with a high versus low capacity for empathy (and the association to a given outcome of interest) to approximate causal mechanisms. Further, we also point out that we conducted multiple mediation models. While the indirect effects are similar (approximately 0.47–0.48), suggesting consistency across exposures and models, there is a risk that these effect sizes are inflated. Other limitations relate to our outcomes. Specifically, our measure for loneliness was created for the study and is thus not validated. For these reasons, we advise caution when interpreting our results. We also note the absence of an explicit measure of vicarious stress. Nonetheless, we argue that the PSS‐10 still represents a suitable proxy measure as past research has found that assessments of vicarious stress are often collinear with general stress scales such as the PSS‐10 (Rauvola et al., 2019). Finally, we also highlight a potential lack of external validity in our results. Our sample comprised a relatively homogeneous sample of university students with only little variation in age (*M* = 24, *SD* = 2.63) and an overrepresentation of women. We also lacked data on other socio‐demographics and sample characteristics (e.g., SES, culture, social connectedness, rural/urban residence, etc.) which might be associated with empathy levels and/or how it is expressed (e.g., in terms of FS, PT, EC, PD).

## 5 CONCLUSION

In the present study, we investigated the extent to which empathy, defined in multifaceted terms, was associated with stress in a sample of Danish university students during the COVID‐19 pandemic. While previous studies have connected empathy with a number of positive individual and interpersonal benefits in a variety of settings, including healthcare, education, workplace environments, and community and social services, we found positive associations between FS, EC, and PD (but not PT) with (vicarious) stress. These associations were mediated by loneliness. On the basis of these results, we argue that conceptualizing and operationalizing empathy in multidimensional terms provides a more complete and nuanced understanding of this construct and its association with mental health outcomes. This, in turn, may help inform empathy initiatives and interventions that aim to facilitate more supportive and compassionate environments in a variety of contexts and settings.

## AUTHOR CONTRIBUTIONS

**Anders Larrabee Sonderlund**: Conceptualization; investigation; writing—original draft; writing—review and editing; visualization; methodology; formal analysis. **Sonja Wehberg**: Conceptualization; investigation; writing—review and editing; visualization; methodology; formal analysis. **Elisabeth Assing Hvidt**: Conceptualization; investigation; writing—review and editing; visualization; methodology; formal analysis.

## CONFLICT OF INTEREST STATEMENT

The authors declare no conflicts of interest.

## FUNDING INFORMATION

This research received funding from the Independent Research Fund Denmark, Grant number: 8108‐00021B.

### PEER REVIEW

The peer review history for this article is available at https://publons.com/publon/10.1002/brb3.70049.

## Data Availability

Data available on request due to privacy/ethical restrictions.
